# Integrin α5β1 mediates the inhibitory effects of vasoinhibin on angiogenesis and vascular permeability

**DOI:** 10.1016/j.jbc.2025.110978

**Published:** 2025-11-25

**Authors:** Magdalena Zamora, Carmen Clapp, Gonzalo Martínez de la Escalera, Juan Pablo Robles

**Affiliations:** 1 Instituto de Neurobiología, Universidad Nacional Autónoma de México (UNAM), Querétaro, Querétaro, México; 2 VIAN Therapeutics, San Francisco, California, USA

**Keywords:** vasoinhibin, integrin α5β1, HGR motif, 16K prolactin, vasopermeability, extracellular matrix, adhesion, endothelial cell, apoptosis, cell surface receptor

## Abstract

Vasoinhibin (Vi) exerts potent inhibitory effects on angiogenesis and vascular permeability through a minimal three-amino acid sequence, the HGR motif. However, the nature of the Vi receptor has remained controversial. Here, we identify integrin α5β1 as the endothelial cell-surface binding molecule mediating the actions of the HGR motif. Vi binds to α5β1 integrin through this motif, and silencing the integrin α5 subunit abolishes the Vi-mediated inhibition of endothelial cell proliferation, invasion, permeability, and tube formation *in vitro*. Likewise, an antibody against integrin α5β1 prevented the antiangiogenic activity of Vi in the Matrigel plug assay *in vivo*. Notably, the HGR motif activates integrin α5β1, as reflected by an increase in endothelial cell adhesion to fibronectin, the canonical ligand of integrin α5β1. These findings identify integrin α5β1 as the molecular target of Vi mediating its antiangiogenic and antivasopermeability actions. Furthermore, a novel integrin activation mechanism leading to suppressed angiogenesis is unveiled, thereby challenging the conventional integrin inhibition approach as a therapeutic intervention.

Angiogenesis, the formation of new blood vessels, is essential during development and becomes largely quiescent in adulthood (1). In pathological conditions, such as cancer and diabetic retinopathy, angiogenesis becomes exacerbated and directly contributes to disease progression (2). Angiogenesis results from the complex interplay of diverse molecular and cellular pathways (3), involving stimulators, such as vascular endothelial growth factor (VEGF) (4), and inhibitors, including peptide hormones (5, 6, 7). Notably, many of these inhibitors are proteolytic fragments of proteins not originally associated with angiogenesis (8), such as vasoinhibin (Vi).

Vi is a naturally occurring fragment of the hormone prolactin that potently inhibits angiogenesis and vascular permeability by suppressing angiogenic signaling pathways, including those triggered by VEGF (9, 10). Beyond its antiangiogenic effects, Vi promotes fibrinolysis, endothelial cell apoptosis, and inflammation (10). The molecular mechanisms that enable Vi to exert its diverse biological effects remain poorly understood. Previous studies have identified two distinct membrane molecular partners for Vi: the multimeric complex formed by plasminogen activator inhibitor-1 (PAI-1), urokinase plasminogen activator (uPA), and urokinase-type plasminogen activator receptor (uPAR), which was proposed to mediate its antiangiogenic and fibrinolytic properties (11); and integrin α5β1, associated with its proapoptotic effects (12). These findings imply that different structural determinants within Vi mediate its binding to these specific molecular partners.

We recently identified two distinct functional motifs in the Vi molecule: the HGR and the HNLSSEM motifs. However, the biological effects of these structural determinants do not align with the actions of the previously reported molecular binding partners of Vi. Although the HNLSSEM motif mediates Vi binding to PAI-1, this interaction promotes fibrinolysis, inflammation, and apoptosis, rather than inhibition of angiogenesis and vascular permeability (13). Moreover, while the HGR motif potently inhibits angiogenesis and vascular permeability (14), it does not bind PAI-1 (13). These discrepancies raised the need to explore the possibility that integrin α5β1 may be the molecular target for the HGR motif to inhibit angiogenesis and vascular permeability.

Integrin α5β1 belongs to a large family of heterodimeric cell-surface receptors composed of α and β subunits, which mediate adhesion to the extracellular matrix and activate signaling pathways regulating cell growth, migration, and survival (15). Integrin α5β1 and its canonical ligand, fibronectin, are particularly critical for angiogenesis, as their coordinated expression in endothelial cells forms a provisional extracellular matrix necessary for vessel formation (16, 17, 18). Specifically, α5β1 integrin recognizes the RGD and PHSRN motifs in fibronectin, facilitating endothelial cell anchoring and triggering signaling cascades leading to the angiogenesis process (17, 19, 20). Despite the essential role of this integrin in angiogenesis, its therapeutic blockade has shown limited clinical success, primarily because of unexpected agonist-like activities exhibited by some antagonists and the simplistic assumption that the mere integrin inhibition of α5β1 should be therapeutically sufficient (21, 22). These challenges have underscored the complexity of integrin-targeted therapies and emphasized the need for additional therapeutic approaches beyond simple integrin disruption.

This work demonstrates that the HGR motif binds to integrin α5β1 and that this interaction mediates the potent inhibition of angiogenesis and vascular permeability exerted by Vi. Furthermore, binding to the HGR motif did not inhibit integrin α5β1-mediated adhesion, but instead, it enhanced endothelial cell adhesion to fibronectin. These results encourage the clinical translation of HGR-based peptides and introduce novel integrin-activating approaches, rather than conventional inhibitory strategies, for suppressing angiogenesis.

## Results

### Vi binds to integrin α5β1 through its HGR motif

**Figure 1:**
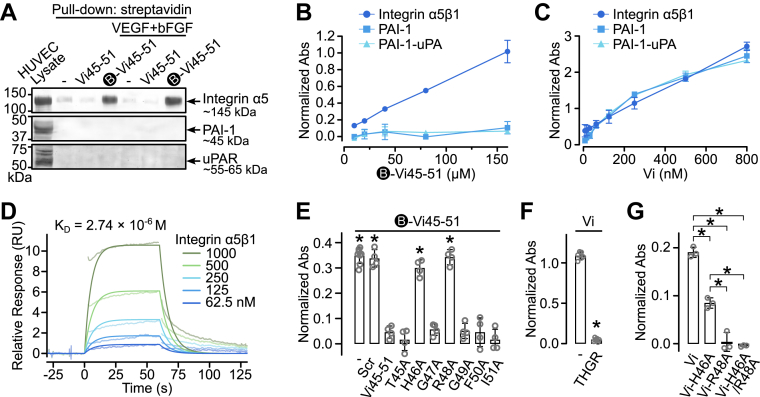
**Binding analysis of the HGR motif to integrin α5β1.***A*, representative pull-down Western blot of HUVEC lysates treated with Vi45-51 or biotinylated Vi45-51 (B-Vi45-51), and VEGF + bFGF, blotted for integrin α5, PAI-1, and uPAR. ELISA evaluating dose-dependent binding of B-Vi45-51 (*B*) or vasoinhibin (Vi) (*C*) to immobilized integrin α5β1, PAI-1, or PAI-1-uPA complex. *D*, SPR analysis of integrin α5β1 binding to immobilized B-Vi45-51 at various concentrations. Curves fitted to a steady-state 1:1 model. *E*, competitive ELISA evaluating B-Vi45-51 binding to immobilized integrin α5β1 with scrambled (Scr) peptide, Vi45-51, or alanine-scanning mutants. ∗*p* < 0.001 *versus* Vi45-51 (ANOVA, Dunnett's test). *F*, competitive ELISA evaluating Vi binding to integrin α5β1 with the HGR-containing tetrapeptide THGR. ∗*p* < 0.001 (unpaired *t* test). *G*, ELISA of Vi and Vi-H46A, Vi-R48A, or Vi-H46A/R48A to integrin α5β1. ∗*p* < 0.001 (ANOVA, Tukey's test). Data are mean ± SD of at least three independent experiments. bFGF, basic fibroblast growth factor; HUVEC, human umbilical vein endothelial cell; PAI-1, plasminogen activator inhibitor-1; SPR, surface plasmon resonance; uPA, urokinase plasminogen activator; uPAR, urokinase-type plasminogen activator receptor; VEGF, vascular endothelial growth factor.

To test whether Vi’s HGR motif binds integrin α5β1, human umbilical vein endothelial cells (HUVECs) were treated with a biotinylated peptide comprising residues 45 to 51 (B-Vi45-51) and subjected to pull-down and Western blot. Integrin α5 was precipitated by B-Vi45-51 but not by Vi45-51 or beads alone, corresponding to a ∼145 kDa protein (Fig. 1*A*). VEGF + basic fibroblast growth factor (bFGF) treatment did not affect binding. B-Vi45-51 failed to pull down PAI-1 or uPAR, or the other described Vi binding partners (11).

An ELISA confirmed dose-dependent binding of B-Vi45-51 to immobilized integrin α5β1 but not to PAI-1 or the PAI-1-uPA complex (Fig. 1*B*). In contrast, Vi bound all three (Fig. 1*C*), with *K*_*D*_ values of 383.2 nM, 527.9 nM, and 405.8 nM, similar to reported affinities (11). Surface plasmon resonance analysis verified direct integrin α5β1 interaction with immobilized B-Vi45-51 (*K*_*D*_ = 2.74 μM) (Fig. 1*D*), consistent with affinities for small peptide motifs (23).

Competitive ELISAs showed that Vi45-51 inhibited B-Vi45-51 binding, whereas a scrambled (Scr) peptide did not. Alanine substitutions at H46 or R48 (H46A or R48A) abolished inhibition (Fig. 1*E*). The tetrapeptide THGR blocked Vi–integrin interaction (Fig. 1*F*), and Vi mutants, Vi-H46A, Vi-R48A, and Vi-H46A/R48A, showed reduced integrin binding (Fig. 1*G*). These results identify the HGR motif as essential for integrin α5β1 recognition.

### Integrin α5β1 is required for the antiangiogenic activity of the HGR motif

**Figure 2:**
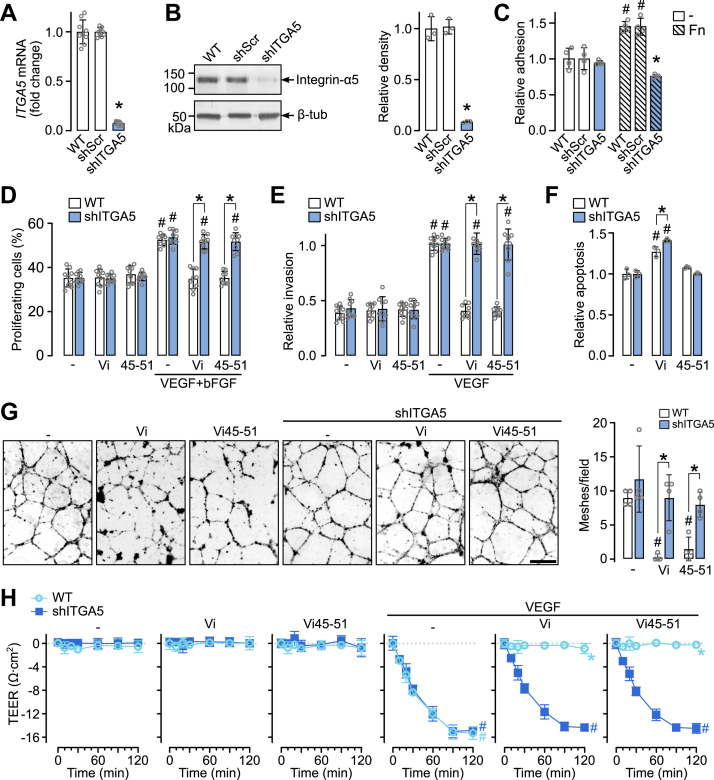
**Effect of integrin α5 knockdown on the antiangiogenic activity of the HGR motif *in vitro*.***A*, expression of *ITGA5* in WT HUVEC or transduced with lentiviral scrambled (shScr) or targeting integrin α5 (shITGA5) shRNA. *B*, representative Western blot and quantification of integrin α5 (molecular weight = 145 kDa) in WT, shScr, and shITGA5 lysates. ∗*p* < 0.001 (ANOVA, Dunnett's test). *C*, adhesion of WT, shScr, or shITGA5 HUVEC to fibronectin (Fn)-coated plates. #*p* < 0.001 *versus* -, ∗*p* < 0.001 *versus* WT. Proliferation (*D*) and Matrigel invasion (*E*) of WT and shITGA5 HUVEC treated with vasoinhibin (Vi) or Vi45-51, stimulated by VEGF + bFGF or VEGF. #*p* < 0.001 *versus* unstimulated cells, ∗*p* < 0.001 *versus* WT. *F*, apoptosis of WT and shITGA5 HUVEC treated with Vi or Vi45-51. #*p* < 0.001 *versus* -, ∗*p* = 0.003 *versus* WT. *G*, representative micrographs of WT and shITGA5 HUVEC tube formation on Matrigel treated with Vi or Vi45-51 and quantification of meshes (closed areas formed by interconnected tubes). #*p* < 0.0017 *versus* -; ∗*p* < 0.008 *versus* WT. The scale bar represents 150 μm. *H*, transendothelial electrical resistance (TEER) of WT or shITGA5 HUVEC monolayers in transwell inserts treated with Vi or Vi45-51 and VEGF. n = 6, #*p* < 0.001 *versus* the absence of VEGF, ∗*p* < 0.001 *versus* WT (repeated-measures ANOVA). Two-way ANOVA, Sidak's test/Dunnett's test. Data are mean ± SD of at least three independent experiments. bFGF, basic fibroblast growth factor; HUVEC, human umbilical vein endothelial cell; VEGF, vascular endothelial growth factor.

To evaluate whether integrin α5β1 is necessary for the antiangiogenic activity of the HGR motif, integrin α5 (*ITGA5*) was silenced in HUVECs using lentiviral-delivered shRNA (shITGA5), reducing 92.5% *ITGA5* mRNA and 91.5% integrin α5 protein (Fig. 2, *A* and *B*). *ITGA5* knockdown reduced 50% HUVEC adhesion to fibronectin (Fig. 2*C*), the canonical integrin α5β1 ligand, but not to an uncoated surface, confirming specific loss of integrin α5β1 function. Since integrin α5 only pairs with β1 (24), other integrins were expected to remain unaffected. The incomplete reduction in fibronectin binding by shITGA5 likely reflects compensation by other integrins (15).

*ITGA5* knockdown abolished the inhibitory effects of Vi and Vi45-51 on endothelial proliferation, invasion, and tube formation (Fig. 2, *D*, *E*, and *G*), demonstrating that integrin α5β1 mediates the antiangiogenic properties of the HGR motif. Furthermore, *ITGA5* silencing ablated the inhibitory effect of Vi and Vi45-51 on the VEGF-induced permeability (Fig. 2*H*). Basal and VEGF + bFGF-stimulated responses were unaffected by *ITGA5* knockdown.

Since it has been previously reported that Vi induces endothelial apoptosis through the integrin α5β1 (12), we evaluated HUVEC apoptosis in *ITGA5*-deficient cells. *ITGA5* knockdown did not abolish Vi-induced apoptosis; in fact, it was even slightly increased (Fig. 2*F*), indicating that apoptosis occurs independently of integrin α5β1. This is consistent with the previous report that the HNLSSEM motif, through uPAR, mediates the apoptosis pathway (13).

**Figure 3:**
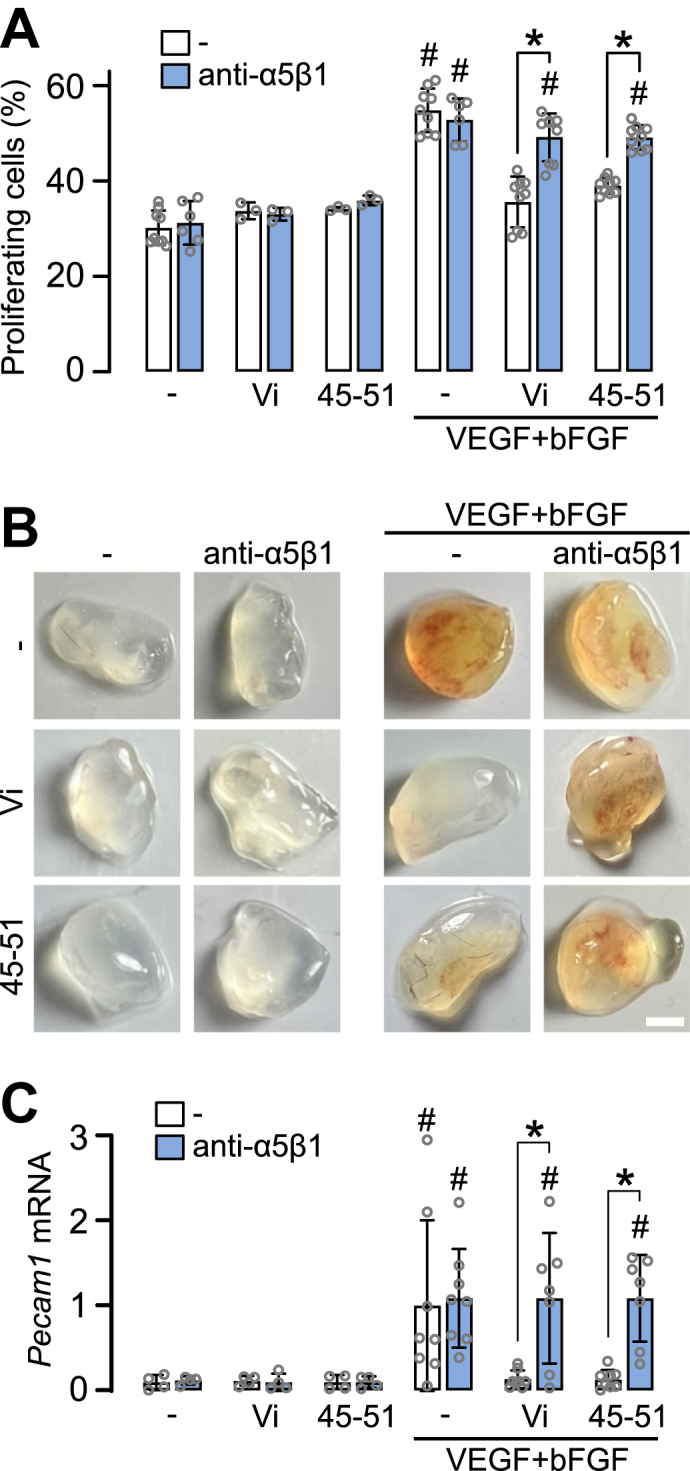
**Effect of anti-integrin α5β1 antibody on the antiangiogenic activity of the HGR motif *in vivo*.***A*, effect of the anti-α5β1 antibody on the inhibitory effect of vasoinhibin (Vi) and Vi45-51 on VEGF + bFGF-induced HUVEC proliferation. #*p* < 0.001 *versus* unstimulated cells, ∗*p* < 0.001 *versus* no anti-α5β1. *B*, representative Matrigel plugs with Vi or Vi45-51, VEGF + bFGF, and anti-α5β1, 7 days after implantation in mice. The scale bar represents 2.5 mm. *C*, quantification of the relative *Pecam1* mRNA expression in the plugs. #*p* < 0.02 *versus* unstimulated plug, ∗*p* < 0.003 *versus* no anti-α5β1. Two-way ANOVA, Sidak's test/Dunnett's test. Data are mean ± SD of at least three independent experiments. bFGF, basic fibroblast growth factor; HUVEC, human umbilical vein endothelial cell; VEGF, vascular endothelial growth factor.

Blocking integrin α5β1 with a nonfunction-blocking antibody (25) also prevented the inhibitory effects of Vi and Vi45-51 on VEGF + bFGF-induced endothelial proliferation (Fig. 3*A*) and Matrigel plug vascularization in mice (Fig. 3*B*), quantified by *Pecam1* (CD-31) (Fig. 3*C*). The anti-α5β1 did not affect basal or stimulated levels, confirming integrin α5β1 as the Vi target in angiogenesis inhibition.

### The HGR motif does not inhibit integrin α5β1–mediated adhesion

**Figure 4:**
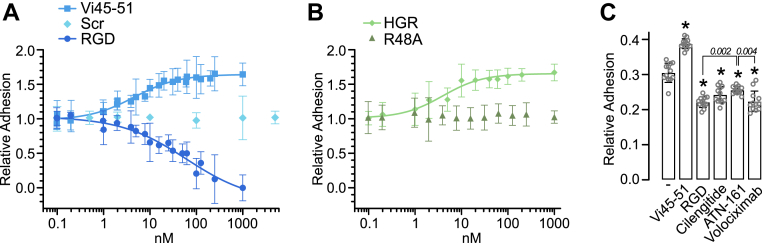
**Effect of the HGR motif on integrin α5β1-mediated endothelial cell adhesion.** Dose–response adhesion of HUVECs onto fibronectin-coated plates in the presence of Vi45-51, scrambled (Scr), RGD (*A*), HGR tripeptide, and Vi45-51 mutated by alanine in R48 (R48A) (*B*). Curves are nonlinear regression fits (*r*^2^ > 0.8). *C*, adhesion of HUVECs treated with 1 μM of Vi45-51, RGD, and integrin inhibitors (cilengitide, ATN-161, and volociximab). ∗*p* < 0.001 *versus* - (ANOVA, Tukey's test). Data are mean ± SD of at least three independent experiments. HUVEC, human umbilical vein endothelial cell; Vi, vasoinhibin.

Since integrin α5β1 inhibition suppresses angiogenesis by disrupting endothelial cell adhesion to the extracellular matrix (17), we tested whether the HGR motif acted similarly. As expected, the RGD tripeptide, which interferes with many integrins (26), inhibited HUVEC adhesion to fibronectin in a dose–response manner (IC_50_ = 61.29 nM). Unexpectedly, Vi45-51 increased the adhesion in a dose-dependent manner (EC_50_ = 4.55 nM) (Fig. 4*A*). An Scr version of the Vi45-51 had no effect. Furthermore, an HGR tripeptide increased adhesion like Vi45-51, but the Vi45-51 carrying an R48A mutation did not (Fig. 4*B*). Unlike known integrin inhibitors, such as RGD-mimetic cilengitide (27), the PHSRN-based peptide ATN-161 (28), and the monoclonal antibody volociximab (29), Vi45-51 enhanced fibronectin-mediated adhesion (Fig. 4*C*), indicating that the HGR motif promotes, rather than blocks, integrin α5β1-dependent adhesion.

## Discussion

**Figure 5:**
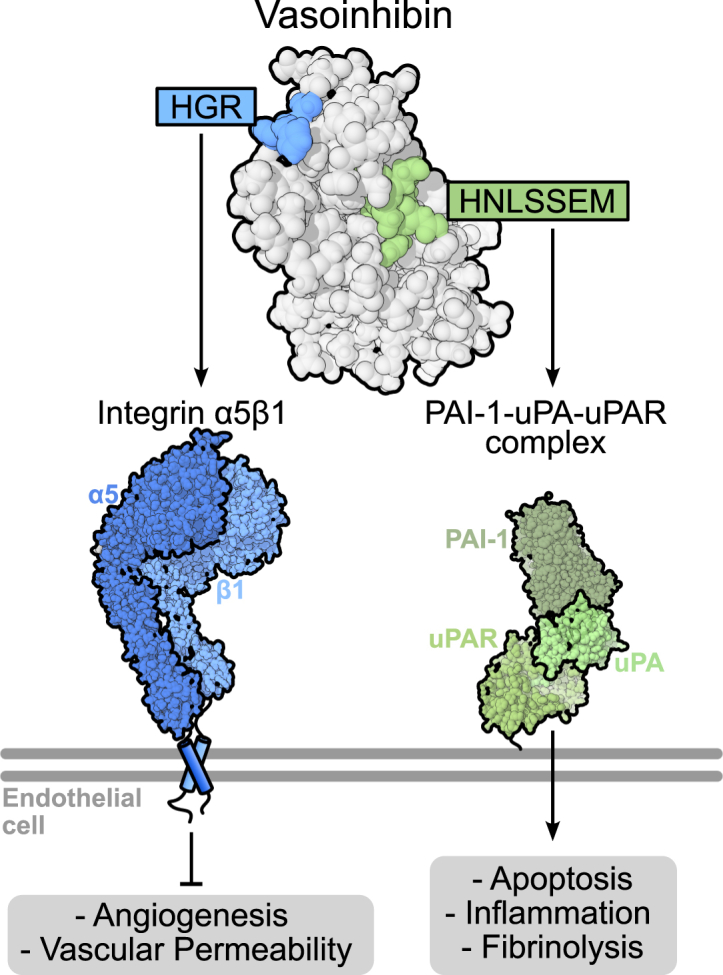
**Vasoinhibin contains two distinct motifs that independently regulate vascular function through specific molecular targets.** The HGR motif of vasoinhibin binds to integrin α5β1 to inhibit angiogenesis and vascular permeability. In contrast, the HNLSSEM motif mediates the binding to PAI-1–uPA–uPAR, inducing endothelial apoptosis, inflammation, and fibrinolysis. PAI-1, plasminogen activator inhibitor-1; uPA, urokinase plasminogen activator; uPAR, urokinase-type plasminogen activator receptor.

Integrins have emerged as promising therapeutic targets because of their significant roles in pathological processes, particularly in exacerbated angiogenesis. However, integrin-targeted therapies have thus far shown limited clinical success (22). In this study, we identified integrin α5β1 as the molecular target responsible for mediating the potent inhibitory effects of the Vi HGR motif on angiogenesis and vascular permeability (Fig. 5). Interestingly, contrary to the prevailing therapeutic paradigm seeking integrin inhibition, the HGR motif activated integrin α5β1 to downregulate angiogenesis, thereby unveiling an alternative modulatory mechanism by which integrin-targeted therapies could operate.

The HGR motif is the sole antiangiogenic determinant of Vi, as its mutation leads to the loss of Vi’s antiangiogenic function (14). Given that Vi binds to integrin α5β1 *via* its HGR motif and that knockdown of ITGA5 abolishes the effects of Vi on endothelial cell proliferation, invasion, vascular permeability, and tube formation *in vitro*, while an antibody against integrin α5β1 suppresses the antiangiogenic effect of Vi in the *in vivo* Matrigel plug assay, integrin α5β1 emerges as the binding partner mediating the antiangiogenic activity of Vi. Integrin α5β1 is critical for angiogenesis (16) and is linked to endothelial cell signaling pathways known to be downregulated by Vi, such as the PI3K/Akt and mitogen-activated protein kinase pathways (10). However, the precise molecular signaling mechanisms downstream of the Vi–integrin α5β1 interaction remain unknown, and our study cannot exclude the contribution of additional molecules, distinct from but influenced by integrin α5β1. Along this line, Vi binds to other endothelial cell surface proteins, specifically the PAI-1–uPA–uPAR multimeric complex (11). The antiangiogenic and antitumor effects of Vi are lost in PAI-1 knockout mice, implying that binding to PAI-1 is essential for Vi’s actions (11). This concept is further supported by reports showing a functional link between integrin α5β1 and uPAR, as their interactions critically modulate endothelial cell migration, extracellular matrix remodeling, and intracellular signaling pathways during angiogenesis (30). Nevertheless, the HGR motif itself does not bind PAI-1 (present data (13)), and peptides containing this motif retain potent antiangiogenic properties without PAI-1 binding (13, 14). Moreover, PAI-1 is known to be proangiogenic (31), and its genetic deletion alone impairs angiogenesis (11). Also, the PAI-1–uPA–uPAR complex lacks intracellular domains to directly transduce antiangiogenic signals (30). And while these observations clearly exclude PAI-1 as the mediator of Vi antiangiogenic properties, binding to PAI-1 *via* the HNLSSEM motif accounts for inflammatory, apoptotic, and fibrinolytic actions of Vi (Fig. 5) and might indirectly influence the regulation of angiogenesis within physiological contexts, such as tissue repair (32), a possibility awaiting investigation.

Given the combinatorial complexity inherent to the different structural determinants of Vi, it is not surprising that different conclusions have been drawn regarding its key biological mediators. Another example is integrin α5β1. Harigaya’s group first described the binding of Vi to integrin α5β1 and showed that antibodies against integrin α5β1 partially prevented Vi-induced endothelial cell apoptosis (12), concluding that integrin α5β1 mediates the apoptotic effects of Vi. In contrast, we found no evidence to support this claim. Silencing integrin α5β1 did not prevent, but slightly enhanced, endothelial cell apoptosis induced by Vi, indicating that integrin α5β1 is not involved in its apoptotic mechanism. This discrepancy may be explained by the fact that certain integrin α5β1 antibodies can actively promote cell survival rather than acting as passive blockers (33). Integrin inhibitors, such as cilengitide (27), RGD, and ATN-161 peptides (28), or the monoclonal antibody volociximab (29), which disrupt endothelial adhesion, induce apoptosis through anoikis (34). However, the HGR motif enhances, rather than disrupts, endothelial cell adhesion to fibronectin, and peptides containing this motif are not proapoptotic (13). Furthermore, our findings align with the previously described HNLSSEM motif of Vi, which induces endothelial apoptosis through a uPAR-dependent mechanism (13). Altogether, these observations support the conclusion that the integrin α5β1 is not the mediator of the apoptotic properties of Vi.

The unexpected observation that the HGR motif enhances, rather than inhibits, endothelial cell adhesion to fibronectin represents a regulatory mechanism distinct from conventional integrin-blocking strategies. Integrin-mediated adhesion typically promotes the proliferation and survival of endothelial cells (35, 36), which is why inhibitory therapeutic compounds (*e.g.*, cilengitide, ATN-161, volociximab, endostatin, and tumstatin) have been developed as antiangiogenic therapies (27, 28, 29). However, under certain contexts, integrin-mediated adhesion may inhibit cell proliferation and migration. For instance, the absence of β3, β5, or α3β1 integrins in endothelial cells enhances angiogenesis (37, 38). Moreover, increased cell adhesion may hinder proliferation, as endothelial cells forming capillary tubes in fibronectin-rich matrices exhibit growth arrest (39). Further evidence of integrin-mediated growth suppression emerges from oncology research. Overexpression of integrin α5β1 can reduce the proliferation of cancer cells (40, 41, 42, 43, 44), and cancer cells that acquire anchorage-independent proliferation typically downregulate integrin α5β1 (45). This functional duality underscores the complexity inherent to integrin regulation of cellular processes.

The mechanism by which increased integrin α5β1-mediated endothelial adhesion suppresses angiogenesis warrants further investigation. Given the notable difference between the potencies of the HGR motif to induce adhesion (EC_50_ ∼4.5 nM) and to inhibit proliferation (IC_50_ ∼150 pM) of endothelial cells, with a relatively low *K*_*D*_ (2.74 μM), it suggests that these effects might not only result from mechanical cues, such as cellular shape, tension, motility, or matrix stiffness (46). Instead, this disparity suggests the involvement of finely tuned signaling amplification pathways downstream of integrin α5β1 activation. Potential mechanisms might include modulation of Rho/Rac signaling components (47), blockage of kinases that regulate cell-cycle progression (43), downregulation of critical growth factor receptors (44), or lateral inhibitory interactions with other cell surface receptors (48).

In conclusion, this study identifies integrin α5β1 as the molecular target for the antiangiogenic effects of the Vi HGR motif and highlights a promising novel mechanism of integrin modulation. These insights advance our understanding of integrin biology and may open therapeutic avenues beyond conventional integrin-inhibitory strategies.

## Experimental procedures

### Materials

Vi45-51, B-Vi45-51, Scr, HGR, THGR, alanine-scanning mutant peptides, and VEGF (GenScript); RGD, cilengitide, ATN-161, and anti-integrin α5 (Sigma–Aldrich); volociximab (Novus Biologicals); recombinant human integrin α5β1 and anti-uPAR (Bio-Techne), fibronectin (Gibco); Matrigel (Corning), bFGF donated by Scios, Inc; anti-β-tubulin and anti-PAI-1 (Abcam); and non–function-blocking anti-α5β1 antibody (clone HA5, MAB1999; Millipore). Vi (49) and its mutants were produced as reported (14). HUVECs were cultured as reported (14, 50).

### Pull-down assay and Western blot

HUVECs treated with 500 nM B-Vi45-51, 20 ng mL^−1^ bFGF, and 25 ng mL^−1^ VEGF for 1 h were lysated in radioimmunoprecipitation assay, sonicated, incubated 30 min at 37 °C, and pulled down with Dynabeads Streptavidin T1 (Thermo). Eluates were analyzed by a Western blot for integrin α5, PAI-1, and uPAR.

### ELISA

Plates coated with 2 μg mL^−1^ integrin α5β1, PAI-1, or PAI-1-uPA complex (37 °C, 10 min) were incubated with B-Vi45-51, Vi, or 100 nM ViH46A, ViR48A, or ViH46A/R48A. Competition used excess Src, alanine mutants, or THGR peptides. Detection used horseradish peroxidase antibodies and 3,3′,5,5′-tetramethylbenzidine.

### Surface plasmon resonance

B-Vi45-51 (100 nM) was immobilized on a streptavidin SA chip (Biacore 1K; Cytiva) at 10 μl min^−1^. Integrin α5β1 was injected (30 μl min^−1^, 60 s association/dissociation) in HBS-P (HEPES-buffered saline solution with a nonionic surfactant) buffer (150 mM NaCl, 1 mM MnCl_2_, pH 7.4). Kinetics were fitted to a 1:1 binding model. Chip was regenerated with running buffer.

### shRNA-mediated integrin α5 knockdown

HUVECs were transduced with pLKO.1-based MISSION shRNA vectors targeting ITGA5 (TRCN0000029652) (NM_002205; Sigma) or Scr #1864 (Addgene).

### Endothelial cell proliferation

As reported (14), HUVECs were serum-starved for 8 h and treated with 100 nM Vi45-51 or Vi, bFGF + VEGF, and 20 μg mL^−1^ anti-α5β1 antibody in 20% fetal bovine serum for 24 h. EdU incorporation was detected with Azide Fluor 545 (Sigma).

### Invasion

As reported (14), HUVECs on Matrigel-coated Transwells were treated with 100 nM of Vi45-51 or Vi. After 16 h, invading cells were counted.

### Apoptosis

As reported (13), HUVEC were treated with 100 nM Vi or Vi45-51 for 24 h, and apoptosis was analyzed using the Cell Death Detection ELISA kit (Roche).

### Endothelial permeability

HUVECs were grown to confluency on 0.4 μm pore Transwells, treated with 100 nM Vi45-51 or Vi for 1 h, then stimulated with 50 ng mL^−1^ VEGF. Transendothelial electrical resistance was recorded for 120 min using the EVOM2 meter (World Precision Instruments).

### Tube formation

HUVEC tubular network formation was evaluated as reported (14, 51). HUVECs were seeded on Matrigel and treated with 100 nM Vi or Vi45-51 for 6 h. Micrographs were analyzed by Angiogenesis Analyzer (52).

### Endothelial adhesion

Ninety-six–well plates, coated with fibronectin (1 μg cm^−2^), were blocked with 20% fetal bovine serum-F12K and incubated with Vi45-51, HGR, R48A, Scr, or RGD, cilengitide, ATN-161, or volociximab. HUVECs (43,000 cells cm^−2^) were added and incubated at 37 °C for 1 h. Cells were washed twice and quantified by 3-(4,5-dimethylthiazol-2-yl)-2,5-diphenyltetrazolium bromide.

### Matrigel plug assay

As previously described (14, 53, 54), 6-week-old male C57BL/6 mice were subcutaneously injected with two 0.5 ml plugs supplemented with 300 ng mL^−1^ bFGF and 100 ng mL^−1^ VEGF, 100 nM Vi or Vi45-51, and 20 μg mL^−1^ anti-integrin α5β1 antibody. After 7 days, plugs were collected, and *Pecam1*, relative to *GAPDH*, was quantified by quantitative PCR as reported (14). Procedures followed institutional ethical approval (Bioethics Committee, Instituto de Neurobiología, UNAM) and were in accordance with the Guide for the Care and Use of Laboratory Animals (http://www.ncbi.nlm.nih.gov/books/NBK54050/).

### Statistical analysis

Statistical analysis was performed using GraphPad Prism 10.5.0 (GraphPad Software, Inc), with a *p* < 0.05 significance threshold.

## Data availability

All data generated or analyzed during this study are included in this article.

## Conflict of interest

J. P. R., M. Z., G. M. E., and C. C. are the inventors of the patent application (WO/2021/098996), which is partially owned by UNAM. J. P. R. is the founder, and M. Z. and C. C. are advisors of VIAN Therapeutics, Inc.
